# Factors Affecting Intention to Disclose HIV Status among Adult Population in Sarawak, Malaysia

**DOI:** 10.1155/2018/2194791

**Published:** 2018-08-15

**Authors:** Aren Sinedeh Lemin, Md Mizanur Rahman, Cliffton Akoi Pangarah

**Affiliations:** Department of Community Medicine and Public Health, Faculty of Medicine and Health Sciences, Universiti Malaysia Sarawak, Kota Samarahan, Malaysia

## Abstract

**Background:**

Disclosure of HIV-positive status is an essential prerequisite for the prevention and care of person living with HIV/AIDS as well as to tackle hidden epidemic in the society.

**Objective:**

To determine the intention to disclose the HIV/AIDS status among adult population in Sarawak, Malaysia, and factors affecting thereof.

**Methods:**

This cross-sectional community-based study was conducted among adult population aged 18 years and above in Sarawak, Malaysia. A gender-stratified multistage cluster sampling technique was adopted to select the participants. A total of 900 respondents were successfully interviewed by face-to-face interview using interview schedule. Stepwise binary logistic regression models were fitted in SPSS version 22.0 to identify the factors associated with the disclosure of HIV/AIDS status. A *p* value less than 0.05 was considered as statistically significant.

**Results:**

The mean (SD) age of male and female respondents was 41.57 (13.45) and 38.99 (13.09) years, respectively. A statistically significant difference of intention to disclosure of HIV status was found between males and females (*p* < 0.05). A stepwise binary logistic regression analysis revealed that age, occupation, knowledge on HIV transmission, and content of discussion about HIV/AIDS appeared to be potential predictors for male respondents to disclose HIV status, while ethnicity and content of discussion on HIV/AIDS were found to be important predictors among the female respondents (*p* < 0.05).

**Conclusion and Recommendation:**

Though the study did not depict the national prevalence of disclosure of HIV/AIDS status, the findings of the study would provide an important basic information for programme intervention, policy, and future research agenda.

## 1. Introduction

Global HIV statistics reported that there were almost 36.7 million people living with HIV of which 1.8 million people became newly infected by HIV in 2016. However, cumulatively, about 76.1 million people were living with HIV and 35.0 million people died from AIDS-related illness since last epidemic [1]. In Malaysia, the total reported HIV/AIDS cases for three decades (1986–2016) were 111,916 and total reported AIDS-related deaths (1986–2016) were 18,827 [2, 3]. In Sarawak, there were 2,178 HIV infections with 480 AIDS-related deaths. The notification rate in Sarawak was 8.7 per 100,000 persons, which was lower than the national average of 10.9 per 100,000 per person [2, 3].

Disclosure of HIV status was an important variable, as disclosure of HIV status to a sexual partner, close relatives, and friends might benefit people living with HIV/AIDS (PLWHA), partners, and society. Some of the potential benefits were improving emotional and psychological well-being [4, 5], early enrolment on antiretroviral therapy, and better adherence to therapy [6, 7], and disclosure to sexual partner may increase HIV testing [4, 8] and reducing risk of HIV transmission [9, 10] including HIV transmission from the mother to child [8, 11].

However, the pattern of disclosure of HIV status varies among community, such as disclosure to sexual partner (56 to 81%), family members (70 to 87%), and friends (26 to 88%) in the United States [12, 13], and disclosure to sexual partner (70%), family members (78%), and friends (7%) in Asia, such as China [14] and India [15]. Besides, pattern of disclosure also varies between genders [16] and it is influenced by various factors, namely, sociodemographic factors [17–19], socioeconomic factors [20], knowledge on HIV [21], and skill of communication to disclose [22].

There is insufficient study available in Malaysia, particularly Sarawak, and this study aimed at determining the willingness to disclose HIV status among adult community in Sarawak, if they are found positive.

## 2. Materials and Methods

### 2.1. Study Design and Sampling

This was a cross-sectional study conducted in Sarawak, Malaysia, for a duration of two years from year 2016 to 2017. A gender-stratified multistage cluster sampling technique was adapted. For the sampling procedure, the Sarawak state was divided into three zones, namely, the northern, southern, and central zone. From each zone, a division was randomly selected followed by two districts selected randomly from each division. From each district, five villages were selected randomly. Then, 30 households were selected by a stratified systematic random sampling where adult males and females aged 18 years and above were selected at every *k*th number of household in the village household list given by the “*Ketua Kampung*” (village headman).

Sample size was calculated with base proportion of stigma and discrimination of 60% [23] with 1.96 standard value for two-tailed tests and 5% absolute precision. The sample size was further inflated by multiplying design effect (2) and 20% nonresponse rate. Thus, the final sample was 885 with rounding into 900. Thirty respondents were recruited from each village.

In this study, inclusion criteria for respondents were adults aged 18 years and above, physically healthy and not being diagnosed with HIV/AIDS, and Malaysian citizens agreeing to participate and able to understand Malay or English. Failure to be interviewed after three attempts was excluded.

### 2.2. Data Collection Instruments and Data Collection Procedure

We developed a data collection instrument after studying with past studies [24–26]. The first part consisted of independent variables of sociodemographic, socioeconomic, and sociocultural characteristics (total 10 items). Then, second part of independent variable were questions on HIV/AIDS transmission-related knowledge. This was measured by 17 items having two key domains, namely, modes of HIV transmission (10 items) and misconception about HIV transmission (7 items) [26]. The score posed true and false answers that were scored one point for each correct answer [26]. The third part of independent variables were questions on communication and discussion about HIV/AIDS [25]. These questionnaires consisted of types of communication (mass media or interpersonal communication) and frequency of communication and content of discussion. Content of discussion consisted of “what were useful information about HIV/AIDS that they would like to know and discuss?” Participants would answer “yes” and “no” accordingly to each question such as on “HIV/AIDS signs and symptoms, treatment of HIV/AIDS, mode of transmission of HIV, HIV prevention methods, and health-related complication of HIV/AIDS.” Then, content of discussion was further divided into poor, moderate, and extensive according to score imposed correct answer. Finally, the fourth part of questionnaire included disclosure of HIV status as dependent variable adapted and modified from Banteyerga et al. [24]. For disclosure of HIV status, respondents responded to “yes” and “no” answer. Then, a sum score for this scale was calculated, for example, “not disclose” to anyone equal to 0 and to disclose to other people equal to 1. Therefore, respondents who answered “yes” to disclose HIV status to partner, parents, family, neighbour, employer, friends, religious leader, and others were scored with 1 for each answer.

### 2.3. Pretest and Quality Control

A pretest of questionnaire was conducted among 30 respondents in a nonsampled area. The purpose of this was to test the consistency, understandability, and flow of questions. Moreover, the data quality is believed to be high as the interviewers were thoroughly trained for one-week, close supervision of the interviewers during data collection and the questionnaires were thoroughly edited to make sure that relevant questions have been responded to and coded according to the code designed for the study. Three attempts were made to get the sampled respondents.

### 2.4. Data Entry and Statistical Analysis

Data coding and verification of response were made on the same day immediately after interview. Any missing information was corrected on the following day to get the correct information. The cleaned data were entered into the computer using SPSS version 22.0 platform [27]. For descriptive statistics, frequency, mean, and standard deviation were presented. For inferential statistics, stepwise binary logistic regression model was fitted to find the associated factors for disclosure of HIV/AIDS status. A *p* value less than 0.05 was considered as statistically significant.

### 2.5. Ethical Issues

Ethical approval was obtained from the Medical Ethics Committee of Universiti Malaysia Sarawak (UNIMAS/NC-21.02/03–02 Jld.2 (08)) dated on 11 February 2016, Clinical Research Centre, and the National Medical Research Register, Ministry of Health (NMRR-16-192-29374 (IIR)) dated on 31 March 2016. All the respondents were briefed about the objectives of the study, and a written informed consent was obtained before data collection.

## 3. Results

### 3.1. Sociodemographic Characteristics

A total of 900 respondents (450 males and 450 females) from 30 villages in Sarawak participated in this study. The mean age of males was 41.57 years and that of females was 38.99 years, and the mean age difference between males and females was statistically significant (*p* < 0.05). However, no statistically significant difference was found in ethnicity, religion, living status, and family size (*p* > 0.05). A statistically significant difference was found in terms of level of education, occupation, and monthly household income (*p* > 0.05) between males and females, indicating the proportion of nonformal education and unemployed was found to be high among the female respondents (Table 1).

### 3.2. Disclosure of HIV

Two-thirds (68.4%) of the respondents desired to disclose if he or she had HIV positive. The highest percentage of the respondents wanted to disclose the status to their partner (56.8%) followed by parents (52%), family (42.4%), and friends (18.4%). However, 14.2% had no choice or preference of person to be disclosed (Table 2).

### 3.3. Gender-Stratified Disclosure of HIV/AIDS Status

Analysis revealed that among the males, the percentage of disclosure was 66.7% compared with females (70.2%) and the difference was statistically significant (*p* < 0.05). However, the Phi coefficient indicated small effect (Table 3).

### 3.4. Gender-Stratified Factors Affecting the Disclosure of HIV Status: Stepwise Binary Logistic Regression Analysis

To identify the potential factors that predict the disclosure of HIV status, stepwise binary logistic regression analysis was done. The variables, namely, age, ethnicity, occupation, knowledge on HIV transmission, and content of discussion on HIV/AIDS, were found to be statistically significant in preliminary analysis by Pearson's chi-square test of independence and were entered into the regression model. The dependent variable was dichotomous into “yes” and “no.” A forward stepwise method was selected to identify potential factors that predict disclosure of HIV status in both genders.

#### 3.4.1. Male Respondents

From bivariate analysis, age, ethnicity, occupation, knew someone had HIV, knowledge on HIV transmission, and content of discussion of HIV/AIDS appeared as significant influencing factors for predicting disclosure of HIV-positive status (*p* < 0.05). However, after binary logistic regression analysis, there were four variables found as important predictors in the final full explainable model (step 4), namely, age in years (40–49 and 50–59), occupation (self-employed), average knowledge of HIV transmission, and moderate content of discussion. The model contains four independent variables which explained 16.2% (Cox and Snell *R* square) and 22.6% (Nagelkerke *R* square) of variance in the disclosure of HIV status. Besides, it was also be able to classify 66.7% of the cases correctly. The goodness of fit was not statistically significant, which showed a good fitted model with homogeneity.

An analysis indicated that age group 40 to 49 years (AOR = 2.253, 95% CI: 1.218, 4.167), age group 50 to 59 years (AOR = 4.686, 95% CI: 2.133, 10.293), occupation as self-employed (AOR = 0.588, 95% CI: 0.347, 0.997), poor knowledge on HIV transmission (AOR = 2.837, 95% CI: 1.294, 6.129), average knowledge on HIV transmission (AOR = 4.582, 95% CI: 2.582, 8.131), and no or poor content of discussion (AOR = 1.890, 95% CI: 1.183, 3.021) and moderate content of discussion (AOR = 4.847, 95% CI: 1.664, 14.118) appeared to be important predictors of disclosure of HIV-positive status. Apart from that, it showed that male respondents aged between 40 and 49 years were 2.253 times likely to be disclosed HIV status compared with those aged less than 30 years. Meanwhile, male respondents aged between 50 and 59 years were 4.686 times likely to be disclosed HIV status, if positive. However, 41.2% of those who worked as self-employed were less likely to be disclosed HIV status compared with those who had gainful job. Apart from that, male respondents who had poor knowledge on HIV transmission were 2.837 times likely to be disclosed HIV status compared with those who had good knowledge on HIV transmission. Similarly, male respondents who had average knowledge on HIV transmission were 4.582 times likely to be disclosed HIV status compared with those who had good knowledge on HIV transmission. Besides, those who had no or poor content of discussion were 1.890 times likely to be disclosed HIV status compared with those who had extensive content of discussion on HIV/AIDS. Similarly, those who had average content of discussion were 4.847 times likely to be disclosed HIV status compared with those who had extensive content of discussion (Table 4).

#### 3.4.2. Female Respondents

Among the female samples, ethnicity and content of discussion of HIV/AIDS appeared as statistically significant influencing factors for predicting disclosure of HIV status (*p* < 0.05). Furthermore, there were two variables identified as important predictors in the final full explainable model (step 2), namely, ethnicity (Malay, Iban, and Bidayuh) and moderate number of issues of discussion on HIV/AIDS. The model has one independent variable which explained 9.2% (Cox and Snell *R* square) and 13% (Nagelkerke *R* square) of variance in the disclosure of HIV status. Besides, it is also be able to classify 72.2% of the cases correctly. The goodness of fit was not statistically significant, which indicated a good fitted model. An analysis showed that Malay ethnicity (AOR = 6.184, 95% CI: 2.921, 13.090), Iban ethnicity (AOR = 2.917, 95% CI: 1.722, 4.943), and Bidayuh ethnicity (AOR = 4.468, 95% CI: 2.211, 9.027) and moderate number of issues of discussion (AOR = 4.317, 95% CI: 1.212, 15.371) appeared to be important predictors of HIV disclosure. Malay female respondents were 6.184 times more likely to be disclosed HIV status, if positive compared with other ethnicities. Similarly, Iban respondents were 2.917 times likely to be disclosed HIV status, if positive compared with others. Bidayuh respondents were 4.468 times more likely to be disclosed HIV status compared with others. Apart from that, those respondents who had moderate number of issues of discussion were 4.317 times likely to be disclosed HIV status compared with those who had extensive discussion on HIV/AIDS (Table 4).

The contents of discussion of HIV/AIDS issues appeared to be important predictor in both male (AOR = 4.847, 95% CI = 1.664, 14.118) and female (AOR = 4.317, 95% CI = 1.212, 15.371) respondents (*p* < 0.05) where moderate number of issues on HIV/AIDS matters encouraged them to disclose HIV status. However, among males, even poor or less number of issues encouraged them to disclose HIV status (AOR = 1.890; 95% CI: 1.183, 3.021).

## 4. Discussion

In our study, the intention to disclose HIV-positive status among respondents was 56.8% to their sexual partner, 52.0% to parents, 42.4% to family members, and 18.4% to friends. This finding indicated that most of them were willing to disclose HIV status if positive to sexual partner and this is similar to other studies in Africa [8]. A possible explanation was there need to be good trust and close relationship to encourage disclosure of HIV status to partners, including family [28]. Apart from that, pattern of disclosure of HIV status was higher among females compared with males (70.2% versus 66.7%). This finding is similar to others in South Africa [29]and the United States [30]. The possible explanation might be that men tend to avoid communication such as HIV disclosure and perceived that was highly personal information and want to protect family from shame [31]. In contrast to this finding, a few studies reported that women are vulnerable to negative complication of HIV disclosure such as blame and physical violence from family and society; therefore, women were afraid to disclose [32–34]. However, some studies found males' pattern of HIV disclosure was higher compared with female counterparts [35–37].

In our study, several factors predicting the disclosure of HIV/AIDS if positive among males were age, occupation, knowledge on HIV transmission, and content of discussion; meanwhile, ethnicity and content of discussion were influencing HIV/AIDS disclosure among female respondents. Male with older age, self-employed, average knowledge on HIV transmission, and moderate discussion on HIV/AIDS increase the disclosure of HIV/AIDS status, whereas females with Malay, Iban, and Bidayuh ethnicity and moderate discussion on HIV/AIDS were associated with disclosure of HIV/AIDS status.

Age was a predictor of willingness to disclose HIV status among male respondents. Those aged 40 to 49 years and 50 to 59 years were more likely to disclose their HIV status if positive compared with others. This indicated older males were more likely to disclose HIV status compared with younger males. This is consistent with other studies [18, 38]. This may be explained that being older, they are likely to have a steady sexual partner, and this contributes to increase the rate of disclosure [38]. Another explanation may be that men are willing to disclose their HIV status if positive, due to their responsibility to disclose due to their concern for their partners' health or to avoid their guilt [39, 40]. This finding also reflected that younger age group may not go for HIV testing, which is consistent with the study by Kabiru et al. [41]. However, Wei et al. [42] did not find any association between age and disclosure of HIV status in Asia.

Ethnicity was one of the predictors for HIV disclosure among females. The current study reported that being Malay, Iban, and Bidayuh, females were more likely to disclose their HIV status if positive. The possible reason was that married or cohabited females were more likely to disclose HIV status to sexual partner due to their intimacy of partners and the confidence they have with each other facilitating open communication and later enhance disclosure of HIV status [43], and furthermore, the majority of female respondents in this study were Iban and married or cohabited with their sexual partners. This is similar to other studies [17, 44] where ethnicity had significant relationship with HIV disclosure. Thus, the issue of disclosure need to be emphasise cultural sensitivity and social challenges among community. Surprisingly, ethnicity was not a predictor for disclosure among males and this needs future study.

Occupation is one of the socioeconomic factors that can influence the disclosure of HIV status among males. This finding was similar to other studies [10, 45], which reported that those who were employed were less likely to disclose their HIV status compared to unemployed. Meanwhile, work as self-employed in the current study is possibly associated with low-wage employment, thus leading to reduce likeliness to disclose their HIV status. This is consistent with the study by Padilla et al. [46]. It also suggests that economic and social disadvantages made disclosure more difficult [47].

Knowledge on HIV transmission was a predictor for disclosure of HIV status among male respondents but not among female respondents. The current study reported that respondents with average knowledge on HIV transmission were 4.5 times more likely to disclose HIV status among males compared with those who had good knowledge on HIV transmission. This is supported by another study [21], which found that willingness to disclose HIV status is negatively associated with misconception of HIV transmission. However, contradictory finding also found that respondents with poor knowledge on HIV transmission were 2.83 times more likely to disclose HIV status among male respondents.

The content of discussion was an important predictor for HIV disclosure among male respondents. This reflected that open discussion and communication on HIV/AIDS among partners and family members particularly encourage the disclosure of HIV status [22, 48]. However, the present study found that moderate content of discussion on HIV/AIDS among both genders was more likely to disclose HIV status. Possible explanation might be that the content of discussion which included HIV information such as HIV testing with family might help individuals to be confident and had good strength mentally and spiritually to disclose to partner because they can accept outcome of their disclosure of HIV status [49]. Another possible explanation might be that the content of discussion includes benefit of disclosure and availability of treatment by health-care worker as doubt about benefits of disclosure is adequately addressed, which may enhance willingness to disclose of HIV status if positive [22].

### 4.1. Limitations of the Study

A few limitations were encountered in the current study. First, the data for this study were collected from rural population; thus, generalisation to the urban population should be done with caution. Second, by nature of cross-sectional study, it is unable to establish causal relationship.

## 5. Conclusion

As a conclusion, this study found that knowledge on HIV transmission and contents of discussion were important issues for disclosure of HIV status, if a person is positive for HIV. Thus, this finding would provide a benchmark and basic information for policymakers and future researchers. Thus, intervention through policymakers should be enhanced through government and nongovernment organization imparting knowledge on HIV transmission and contents of discussion on HIV/AIDS among individual, families, and health-care providers. Besides, researcher may provide evidences to support intervention in order to improve disclosure of HIV among community.

## Figures and Tables

**Table 1:** Sociodemographic characteristics (*n*=900).

Characteristics	Male		Female		*p* value
	Frequency	%	Frequency	%	
^a^ *Age (mean, SD) in years*	41.57 (13.45)		38.99 (13.09)		**0.004**
^b^ *Ethnicity*					
Iban	77	49.0	80	51.0	0.988
Malay	200	50.6	195	49.4	
Bidayuh	73	49.7	74	50.3	
Others	100	49.8	101	50.2	
^b^ *Religion*					
Christianity	106	49.3	109	50.7	0.705
Islam	313	49.8	316	50.2	
Others	31	55.4	25	44.6	
^b^ *Living status*					
Living with partner	329	73.1	325	72.2	0.765
Living without partner	121	26.9	125	27.8	
^c^ *Median family size*	5.0	5.0	0.716		
^b^ *Level of education*					
No formal education	76	36.2	134	63.8	**<0.001**
Primary school	105	55.6	84	44.4	
Secondary school	233	53.9	199	46.1	
Tertiary and above	36	52.2	33	47.8	
^b^ *Occupation*					
Unemployed	63	17.2	303	82.8	**<0.001**
Self-employed	187	75.1	62	24.9	
Government job	61	66.3	31	33.7	
Private job	139	72.0	54	28.0	
^c^ *Median household income (MYR)*	900.0		800.00		**0.005**

^a^ *p* value reached from independent sample *t*-test; ^b^*p* value reached from chi-square test; ^c^*p* value reached from Mann–Whitney *U* test. ^*∗*^*p* < 0.05; ^*∗∗*^*p* < 0.01; ^*∗∗∗*^*p* < 0.001.

**Table 2:** Percentage distribution of respondents by disclosure of HIV status and its pattern.

Variables	*n*	%
*Disclosure of HIV*		
No	284	31.6
Yes	616	68.4
^*∗*^ *Person to be disclosed (n*=616)		
Partner	511	56.8
Parents	468	52.0
Family	382	42.4
Friends	166	18.4
Religious leader	125	13.9
Neighbour	113	12.6
Employer	100	11.1
Others	128	14.2

^*∗*^Multiple responses.

**Table 3:** Gender-stratified percentage distribution of disclosure of HIV/AIDS.

Disclosure of HIV	Male (*n*=450)		Female (*n*=450)		*χ* ^2^ (df)	*p* value	Phi coefficient
	*n*	%	*N*	%			
No	150	33.3	134	29.8	1.31 (1)	0.251	0.038^*∗*^
Yes	300	66.7	316	70.2			

^*∗*^ *p* < 0.05; ^*∗∗*^*p* < 0.01; ^*∗∗∗*^*p* < 0.001. *p* value reached from chi-square test.

**Table 4:** Gender-stratified factors affecting the disclosure of HIV status: binary logistic regression analysis.

Variables	*β*	Male		*β*	Female	
		AOR	95% CI		AOR	95% CI
*Age in years*					NI	
<30 (RC)		1				
30–39	−0.113	0.893	0.465, 1.713			
40–49	**0.812** ^*∗*^	2.253	1.218, 4.167			
50–59	**1.545** ^*∗∗∗*^	4.686	2.133, 10.293			
>60	0.725	2.064	0.907, 4.695			
*Ethnicity*						
Malay				**1.822** ^*∗∗∗*^	6.184	2.921, 13.090
Iban		NI		**1.071** ^*∗∗∗*^	2.917	1.722, 4.943
Bidayuh				**1.497** ^*∗∗∗*^	4.468	2.211, 9.027
Others (RC)					1	
*Occupation*						
Gainful job (RC)		1				
Self-employed	**−0.532** ^*∗*^	0.588	0.347, 0.997		NI	
Unemployed	0.559	1.749	0.946, 3.235			
*Knowledge on HIV transmission*			NI			
Poor (≤6)	**1.043** ^*∗∗*^	2.837	1.294, 6.129			
Average (7–14)	**1.522** ^*∗∗∗*^	4.582	2.582, 8.131			
Good (≥15) (RC)		1				
*Content of discussion*						
No or poor (≤2.30)	**0.637** ^*∗∗*^	1.890	1.183, 3.021	−0.001	0.999	0.624, 1.598
Moderate (2.31–5.13)	**1.578** ^*∗∗*^	4.847	1.664, 14.118	**1.463** ^*∗*^	4.317	1.212, 15.371
Extensive (≥5.14) (RC)		1			1	
Constant	−1.393	0.248		−0.177	0.838	

Model chi-square (df)		79.751 (10)^*∗∗∗*^			43.336 (5)^*∗∗∗*^	
*n*		450			450	
Goodness of fit		9.976 (8); 0.267			6.351 (6); 0.385	
Nagelkerke *R* square		0.226			0.130	
Cox and Snell *R* square		0.162			0.092	

^*∗*^ *p* < 0.05; ^*∗∗*^*p* < 0.01; ^*∗∗∗*^*p* < 0.001. Dependent variable = disclosure of HIV status (yes versus no). RC = reference category; AOR = adjusted odds ratio; NI = not included.

## Data Availability

The data used to support the findings of this study are available from the corresponding author upon request.

## Abbreviations

PLWHA: People living with HIV/AIDS
HIV: Human immunodeficiency virus
AIDS: Acquired immunodeficiency syndrome
AOR: Adjusted odds ratio
CI: Confidence interval
SD: Standard deviation.
